# *Petrocodon rubiginosus*, a new species of Gesneriaceae from Guangxi, China

**DOI:** 10.3897/phytokeys.157.32270

**Published:** 2020-08-26

**Authors:** Rui-Li Zhang, Shu Li, Stephen Maciejewski, Yi-Gang Wei

**Affiliations:** 1 Beijing Engineering Research Center of Rural Landscape Planning and Design, College of Landscape Architecture, Beijing University of Agriculture, Beijing Laboratory of Urban and Rural Ecological Environment, CN-102206, Beijing, China Beijing University of Agriculture Beijing China; 2 Guangxi Key Laboratory of Plant Conservation and Restoration Ecology in Karst Terrain, Guangxi Institute of Botany, Guangxi Zhuang Autonomous Region and Chinese Academy of Sciences, CN-541006 Guilin, China Guangxi Institute of Botany Guilin China; 3 The Gesneriad Society, Inc. 1122 East Pike Street, PMB 637, Seattle, Washington, USA Guilin Botanical Garden Guilin China; 4 Gesneriad Conservation Center of China, Guilin Botanical Garden, Guangxi Zhuang Autonomous Region and Chinese Academy of Sciences, CN-541006 Guilin, China The Gesneriad Society Seattle United States of America

**Keywords:** Jingxi, Karst, Limestone flora, New taxa, Sino-Vietnamese border area

## Abstract

*Petrocodon rubiginosus* Y.G. Wei & R.L. Zhang, **sp. nov.**, from Guangxi of South China, is described and illustrated with photographs. The new species is morphologically similar to *Pet. hechiensis*, but can be easily distinguished by a combination of characters, especially in its petioles, peduncles and pedicels covered with densely ferruginous pilose hairs.

## Introduction

Although the newly delimitation *Petrocodon* Hance is not the most speciose genus in Gesneriaceae of China, the highly variable corolla and leaf morphology impels us to continuously study and understand the species biodiversity of this genus (Lu et al. 2017, Xu et al. 2017). Now, approximately 35 species and one variety range from China to northern Thailand and northern Vietnam (Weber et al. 2011, Chen et al. 2014, Xu et al. 2014, Middleton et al. 2015, Möller et al. 2011, 2013, 2016).

During a floristic expedition to Guangxi, China in 2015, the authors observed a population of an interesting Gesneriaceae in Yuexu Town, Jingxi city, Guangxi. We confirmed that it is a member of the genus *Petrocodon* because it looks like *Pet. hechiensis* (Y.G.Wei, Yan Liu & F.Wen) Y.G.Wei & Mich.Möller (Wei et al. 2008, Weber et al. 2011). Over the past three years, the living plants were monitored in the conservation nursery of the Gesneriad Conservation Centre of China (GCCC) in Guilin Botanical Garden and in the field, where an ecological survey was conducted.

After thorough comparisons of diagnostic morphological and anatomical features of similar taxa from China, Vietnam and Thailand (Wang et al. 1990, 1998, Li and Wang 2005, Phuong 2005, Wei et al. 2010, Middleton et al. 2015), it has been revealed that its morphological characters do not fit any known species, therefore, we conclude that it is a new species to science and accordingly describe it herein. Its morphological characters are compared with the closely related species, *Petrocodon hechiensis*.

## Material and methods

Measurements and morphological character assessments of the new species were performed and described using specimens obtained by the current authors, living material observed in the field and also cultivated at GCCC. All available *Petrocodon* specimens of China, Thailand and Vietnam, stored in the following herbaria, were examined: E, GH, HN, IBK, K, KUN, MO, PE, PH, US and VNMN. At the same time, the specimen’s images and name list of the above-mentioned species were obtained and checked from Tropicos (http://www.tropicos.org), JSTOR Global Plants (http://plants.jstor.org), The Plant List (http://www.plantlist.org/) and the International Plant Names Index (http://www.ipni.org). All morphological characters were studied under dissecting microscopes and are described using the terminology presented by Wang et al. (1990, 1998).

## Taxonomic Treatment

### Petrocodon rubiginosus

Taxon classificationPlantaeLamialesGesneriaceae

Y.G.Wei & R.L.Zhang sp. nov.

825D9EA8-A805-5E14-999F-9D008EB77F1D

urn:lsid:ipni.org:names:77211194-1

Figure 1

#### Diagnosis.

It is morphologically close to *Pet. hechiensis*, but can be distinguished by its petioles, peduncles and pedicels densely curly rubiginous to ferruginous villous, bracts lanceolate to subulate and both surfaces densely rubiginous to dark brown pubescent, corolla lobes oblong to nearly rounded and margin entire, calyx lobes densely rubiginous to ferruginous pubescent.

**Figure 1.**
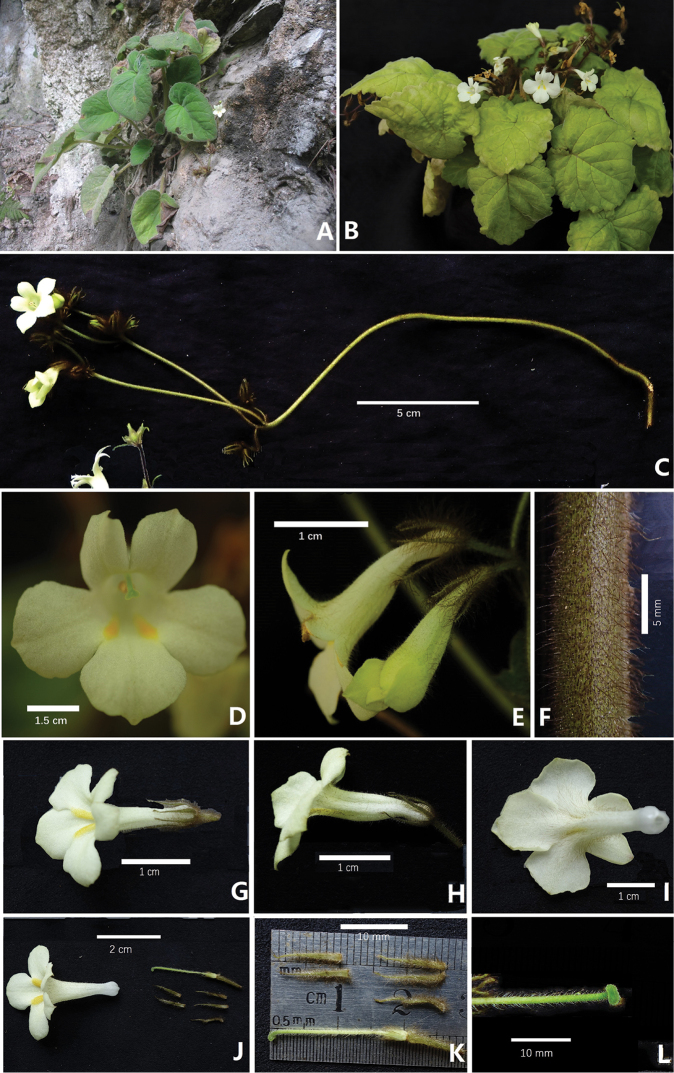
*Petrocodon rubiginosus* Y.G.Wei & R.L.Zhang, sp. nov. **A** flowering plant in natural habitat (Jingxi, Guangxi, China) **B** flowering plant cultivated in GCCC**C** cyme **D** corolla in front view **E** corolla in lateral view and bud on cyme **F** indumentum of peduncle **G** flower in top view **H** flower in lateral view **I** flower in upward view **J** resolved flower for showing corolla, calyx lobes, pistil and pedicel **K** pistil and calyx lobes (adaxial & abaxial surfaces) **L** style and stigma. Photographs by authors.

#### Type.

CHINA. Guangxi Zhuangzu Autonomous Region: Jingxi city, Yuexu Town, Siming village, 22°56'N, 106°37'E, alt. 470 m, 12 November 2015, flowering, *Wen Fang et al*. *WF151112-01* (holotype: IBK; isotype: IBK).

#### Description.

Perennial herb, stemless. *Rhizome* subterete, 3–8 cm long, 5–7 mm in diam. *Leaves* 8–16, all basal; leaf blades herbaceous, green to yellowish-green, zygomorphic to slightly unequal in each pair, broadly ovate to nearly rounded, 3.5–20 × 3.8–15 cm, apex obtuse to rounded, base cordate or obliquely cordate and bases on both sides usually overlapping, margin obviously crenate to undulate, pubescent on both surfaces, 5–6 nerves on each side, adaxially impressed, abaxially prominent, densely ferruginous and erect villous along abaxial main vein and lateral veins; petioles 15–25 cm or longer, densely ferruginous and erect villous. *Cymes* 4–8, 20–30 cm long, usually curved or squiggly,1–2-branched, 8–12-flowered; peduncle 15–20 cm long, ca. 2 mm in diam., densely curly rubiginous to ferruginous villous; bracts 2, opposite, lanceolate to subulate, 5–6 × 1–2 mm, entire, both sides densely rubiginous to dark brown pubescent; bracteoles 2, opposite, linear-lanceolate, ca. 5 ×0.8 mm, both sides densely rubiginous to dark brown pubescent; pedicel 4–7 mm long, 0.8–1 mm in diam., densely rubiginous to dark brown pubescent. *Calyx* 5-parted to the base, sepals lanceolate-linear to subulate, ca. 10 mm long, 0.6–0.7 mm at base, both sides densely rubiginous to dark brown pubescent. *Corolla* bilabiate, pale yellow, throat with two brightly yellow longitudinal stripes and dark yellow glands on stripe surface, ca. 3 cm long, outside white pubescent; tube slender, 2–2.2 cm long, 6.5–7 mm in diam. at middle, slightly constricted at ca. 6 mm above base of corolla base; adaxial lip 7–8 mm long, 2-partite nearly to base, lobes oblong to nearly rounded, apex acuminate, abaxial lip 2–2.5 cm long, 3-partite nearly to base, lobes nearly rounded, apex obtuse. *Stamens* 2, sparsely glandular-puberulent, adnate to 1.1–1.2 cm above the base of corolla tube; *filaments* pale yellow to white, 5–5.5 mm long; *anthers* 1.2–1.5 mm long, elliptical, 1.8–2 mm long, 1–1.5 mm in diam. *Staminodes* 3, glabrous, lateral ones 6–7 mm long, adnate to ca. 10 mm above the base of corolla tube, median ca. 1.5 mm long, adnate to ca. 7 mm above the base of corolla tube. *Disc* glabrous, ca. 1.2 mm high, margin entire. *Pistil* ca. 2 cm long; *ovary* ovoid, ca. 5 mm long, covered pale rubiginous or white pubescent, *style* ca. 1.5 cm long, densely pubescent; *stigmas* inapparent triangular, 2, each one semicircular, ca. 0.8 mm long. *Capsule* ovoid, valvular dehiscence, pubescent.

**Figure 2.**
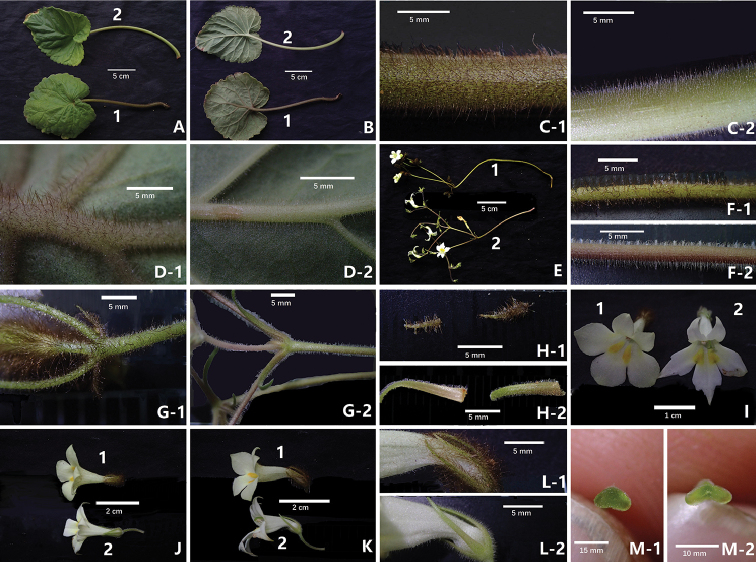
*Petrocodon rubiginosus* Y.G.Wei & R.L.Zhang, sp. nov. (**1**) and its congener, *Pet. hechiensis* (Y.G.Wei, Yan Liu & F.Wen) Y.G.Wei & Mich.Möller (**2**) **A** adaxial surfaces of leaf blades and petioles of two species **B** abaxial surfaces of leaf blades and petioles of two species **C** indumentum of petioles **D** indumentum of main nerves on adaxial surface **E** cymes and flowers **F** indumentum of peduncles **G** indumentum of bracts and pedicels **H** abaxial and adaxial surfaces of bracts **I** corolla in frontal views **J** corolla in top views **K** corolla in lateral views **L** indumentum of abaxial surfaces of calyx lobes **M** stigma. Photographs by authors.

#### Phenology.

Flowering occurs in November and fruiting from December to January of next year.

#### Etymology.

The specific epithet is derived from the conspicuous indumentum of petioles, peduncles and pedicels, which are covered with densely long ferruginous or rubiginous hairs.

#### Vernacular name.

Xìu Géěng Shí Shān Jù Tái (Chinese pronunciation); 锈梗石山苣苔 (Chinese name).

#### Distribution and habitat.

*Petrocodon rubiginosus* is hitherto only known from two close localities at elevational ranges from 450–500 m in Jingxi city, Guangxi, China, growing on shaded and moist rock surface with no more than 50 individuals at each of the localities. Two localities are about three kilometres apart. The species grows in subtropical broad-leaved evergreen monsoon forest with sufficient seasonal run-off water.

#### Preliminary conservation assessment.

Population information of *Petrocodon rubiginosus* is still unclear, which makes it difficult to determine an assessment of the extinction risk faced by this new taxon. At present, two distribution points of the species are known and its estimated area of occupancy is less than 10 km^2^. The main threat now comes from environmental damage caused by grazing and there is a risk of poaching in the future because its distribution is not far away from the villages. Furthermore, prolonged droughts and illegal logging in the area, including nearby potential habitat, should be considered as potential risks to the persistence of *Pet. rubiginosus*. Thus, following the IUCN Red List Categories and Criteria (IUCN 2017), it is assessed temporarily as endangered [EN B2ab (ii, iii)].

#### Notes.

**Figure 3.**
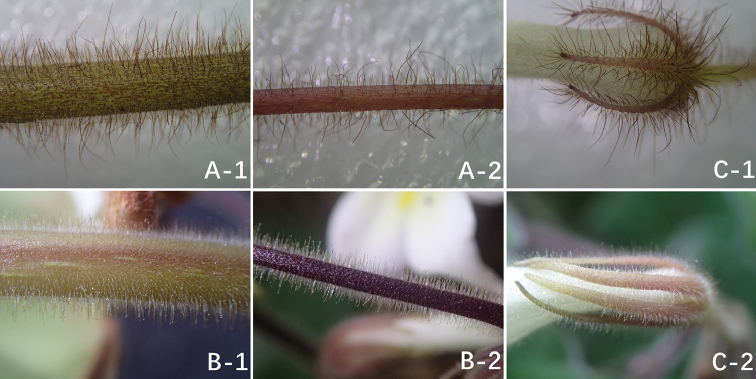
The difference of indumentum between *Petrocodon rubiginosus* Y.G.Wei & R.L.Zhang, sp. nov. (**A**) and *Pet. hechiensis* (Y.G.Wei, Yan Liu & F.Wen) Y.G.Wei & Mich.Möller (**B**): densely curly rubiginous to ferruginous villous on surface of petiole (**A–1**), pedicel (**A–2**) and calyx lobes (**C–1**) and densely short glandular-pubescent and pubescent on surface of petiole (**B–1**), pedicel (**B–2**) and calyx lobes (**C–2**).

It is morphologically close to *Petrocodon hechiensis* because two congeners share some similarities, for example, they have look-alike leaf shape and size and a pale-yellow corolla (Wei et al. 2008, 2010). As a result, it is sometimes mistaken for *Pet. hechiensis*. However, one of the largest differences between *Pet. rubiginosus* and *Pet. hechiensis* is the indumentum of petioles, peduncles and pedicels. There are absolutely no glandular-hairs on petioles, peduncles and pedicels of *Pet. rubiginosus*, but in *Pet. hechiensis*, all are covered by densely short and sticky glandular-hairs and pubescent-hairs (Figure 3). The other major differences between the species are outlined in Table 1.

**Table 1.** Diagnostic character differences between *Petrocodon rubiginosus* sp. nov. and *Pet. hechiensis* (Figure 2).

Characters	*Pet. rubiginosus*	*Pet. hechiensis*
Indumentum of petioles, peduncles and pedicels	densely curly rubiginous to ferruginous villous (C-1, F-1)	densely short glandular-pubescent and pubescent (C-2, F-2)
Bracts	lanceolate to subulate, 5–6 × 1–2 mm, both sides densely rubiginous to dark brown pubescent (G-1, H-1)	linear-lanceolate, 8–16 × 1–2 mm, both sides glandular-pubescent to hispid (G-2, H-2)
Corolla lobes	oblong to nearly rounded, margin entire (I-1)	lanceolate-triangular, margin dentate (I-2)
Calyx lobes	densely rubiginous to ferruginous pubescent (L-1)	densely white glandular-pubescent and pubescent (L-2)
Stigma	inapparent triangular, 2, each one semicircular (M-1)	slightly curved oblong, 2, each one oblong to rounded (M-2)
Florescence	November	September to October

## Supplementary Material

XML Treatment for Petrocodon rubiginosus
